# Physiological consequences of the *P2328S* mutation in the ryanodine receptor (*RyR2*) gene in genetically modified murine hearts

**DOI:** 10.1111/j.1748-1716.2008.01865.x

**Published:** 2008-10

**Authors:** C A Goddard, N S Ghais, Y Zhang, A J Williams, W H Colledge, A A Grace, C L-H Huang

**Affiliations:** 1 Physiological Laboratory, University of CambridgeCambridge, UK; 2 Department of Biochemistry, University of CambridgeCambridge, UK; 3 Department of Cardiology, Wales Heart Research Institute, Cardiff University School of MedicineCardiff, UK

**Keywords:** cardiac action potentials, cardiac arrhythmogenesis, catecholaminergic polymorphic ventricular tachycardia, genetically modified mice, ryanodine receptor, P2328S-RyR2

## Abstract

**Aim:**

To explore the physiological consequences of the ryanodine receptor (RyR2)-*P2328S* mutation associated with catecholaminergic polymorphic ventricular tachycardia (CPVT).

**Methods:**

We generated heterozygotic (*RyR2*^*p/s*^) and homozygotic (*RyR2*^*s/s*^) transgenic mice and studied Ca^2+^ signals from regularly stimulated, Fluo-3-loaded, cardiac myocytes. Results were compared with monophasic action potentials (MAPs) in Langendorff-perfused hearts under both regular and programmed electrical stimulation (PES).

**Results:**

Evoked Ca^2+^ transients from wild-type (*WT*), heterozygote (*RyR2*^*p/s*^) and homozygote (*RyR2*^*s/s*^) myocytes had indistinguishable peak amplitudes with *RyR2*^*s/s*^ showing subsidiary events. Adding 100 nm isoproterenol produced both ectopic peaks and subsidiary events in *WT* but not *RyR2*^*p/s*^ and ectopic peaks and reduced amplitudes of evoked peaks in *RyR2*^*s/s*^. Regularly stimulated *WT*, *RyR2*^*p/s*^ and *RyR2*^*s/s*^ hearts showed indistinguishable MAP durations and refractory periods. *RyR2*^*p/s*^ hearts showed non-sustained ventricular tachycardias (nsVTs) only with PES. Both nsVTs and sustained VTs (sVTs) occurred with regular stimuli and PES with isoproterenol treatment. *RyR2*^*s/s*^ hearts showed higher incidences of nsVTs before but mainly sVTs after introduction of isoproterenol with both regular stimuli and PES, particularly at higher pacing frequencies. Additionally, intrinsically beating *RyR2*^*s/s*^ showed extrasystolic events often followed by spontaneous sVT.

**Conclusion:**

The *RyR2-P2328S* mutation results in marked alterations in cellular Ca^2+^ homeostasis and arrhythmogenic properties resembling CPVT with greater effects in the homozygote than the heterozygote demonstrating an important gene dosage effect.

Cardiac ryanodine receptors (RyR2s) are large tetrameric channels mediating Ca^2+^-induced release of intracellularly stored Ca^2+^ (CICR) into the cytosol following opening of voltage-dependent sarcolemmal L-type Ca^2+^ channels (LTCC) during action potentials (Bers 2002, Wehrens *et al.* 2003, Kontula *et al.* 2005, Yano *et al.* 2005b, Phrommintikul & Chattipakorn 2006). Each (∼565 kDa) RyR2 monomer is associated with a range of accessory, potentially regulatory, proteins. These include calmodulin (CaM), the 12.6 kDa protein FKBP12.6 (also known as calstabin 2), protein kinase A (PKA), protein phosphatase 1 and 2A (PP1 and PP2A), and calmodulin-dependent protein kinase II (CaMKII) bound to its cytoplasmic scaffold domain at the N-terminal region (Bers 2002). It has been suggested that FKBP12.6 binding to RyR2 stabilizes the closed state of the channel during diastole and promotes the coupling of RyR2 subunits enabling channel opening during excitation contraction coupling (Yano *et al.* 2000, 2005a, Bers 2002, Wehrens *et al.* 2004).

The gene encoding RyR2s has been implicated in the autosomal dominant inherited condition, catecholaminergic polymorphic ventricular tachycardia (CPVT), which is associated with presentations of adrenergically (exercise)-induced bi-directional and polymorphic ventricular tachycardia (VT) potentially leading to sudden cardiac death (SCD) in the absence of structural cardiac disease (Laitinen *et al.* 2001, Priori *et al.* 2001, 2002, Wehrens & Marks 2003). It has been suggested that an increased release of SR Ca^2+^ into the cytosol causes delayed diastolic afterdepolarizations (DADs) in the action potential leading to triggered activity in common with findings in digitalis-induced VT (Rosen & Danilo 1980, Leenhardt *et al.* 1995, Tunwell *et al.* 1996, Priori *et al.* 2001, Kummer *et al.* 2006). Thus recent reports have similarly implicated alterations in SR calsequestrin in such pathology (Chopra *et al.* 2007).

Two groups first identified *RyR2* mutations in CPVT patients; neither reported structural cardiac abnormalities or echocardiographic evidence of cardiac failure (Laitinen *et al.* 2001, Priori *et al.* 2001). The first reported four missense mutations of which S2246L, R2427S and N4104K were sporadic and R4497C was found in five clinically affected mutant carriers. The second group demonstrated three unrelated Finnish families carrying mutations in P2328S, Q4201R and V4653F. Seventeen members of the first of these families carried the P2328S mutation. Of these, 10 of the 12 studied showed exercise-induced VT. Two of these P2328S carriers were asymptomatic but showed abnormalities on clinical testing. The carriers within the family showed normal QTc intervals, and mortality rates as reflected in Kaplan–Meier analyses of 30–33% by the age of 35 years in common with the Q4201R and V4653F carriers.

Genetically modified murine hearts are increasingly used as models of human cardiac disease (London, 2001). However, translation of results from isolated murine hearts to the *in vivo* clinical setting must consider differences between both (1) species as well as (2) the electrophysiological assessment protocols employed. Thus (1) despite similar anatomy and fibre organization, murine and human hearts are markedly different in basal heart rate, heart size and ion channel expression (Vaidya *et al.*, 1999). Although murine and human ventricular APs share a steep upstroke reflecting rapid Na^+^ channel gating (Guo *et al.* 1999) and a strong influence of transient outward repolarizing K^+^ current (*I*_to_) on AP recovery, differences in repolarizing currents cause murine ventricular APs to overshoot and fall sharply in contrast to the pronounced plateau phase of the human ventricular AP (Danik *et al.* 2002). (2) A significant proportion of the physiological assay techniques examined isolated perfused as opposed to *in situ* hearts for limited (h) rather than prolonged (years) intervals. Nonetheless, at the very least, murine hearts provide useful resemblances with human hearts the extent of which merits basic investigation. Thus, they have successfully been used to replicate arrhythmogenic properties associated with Brugada (BrS) and LQT3 syndromes (Stokoe 2007a,b; Thomas *et al.* 2007a,b). Similarly, a CPVT-like arrrhythmogenesis has been observed under conditions of adrenaline and/or caffeine perfusion in a recent murine model with RyR2 modifications targeted instead at the C-terminal luminal sites implicated in store-overload-induced Ca^2+^ release (SOICR) (Jiang *et al.* 2004, 2005, Cerrone *et al.* 2005).

This article reports the generation of murine hearts containing P2328S mutations in the central region of the *RyR2* gene close to the FKBP12.6 binding site, and represents the first time both heterozygotic (*p/s*) and homozygotic (*s/s*) transgenic mice are compared and studied. Previous studies in HEK293 cells had associated the P2328S variant with altered FKBP12.6 binding following PKA phosphorylation (Lehnart *et al.* 2004). This variant therefore directly complements genetically modified murine hearts with alterations targeted at the C-terminal luminal sites thought to be involved in SOICR (Jiang *et al.* 2004, 2005, Cerrone *et al.* 2005). The present experiments examined the physiological consequences of both *p/s* and *s/s* mutations not only through cytosolic Ca^2+^ measurements in isolated cardiac myocytes but also electrophysiological recordings in Langendorff-perfused hearts, and the extent to which the intact murine systems show arrhythmogenic properties. Our experiments thus clarified the physiological consequence of the P2328S (*p/s*) and (*s/s*) mutation, and defined the extent to which results from physiological studies demonstrate that this murine system resembled the human phenotype.

## Material and methods

### Generation of RyR2^P2328S^ knock-in mice

The targeting vector for homologous recombination consisted of a 5.89 kb genomic DNA fragment spanning a region from intron 44 to intron 47 of the RyR2 genomic sequence. The 5′ and 3′ flanks were amplified from 129Sv/Ev genomic DNA by PCR with *PfuTurbo* (Stratagene, Amsterdam, the Netherlands) using primers designed to the mouse genome reference sequence ENSMUSG00000021313. The codon change leading to the P2328S variant occurs in exon 45. The 5′ flank was amplified using primers on either side on exon 45 to produce a 965-bp fragment. The forward primer contained an *Avr*II site which could be used to linearize the final targeting construct (5′flankror: GTAAAGAATGAGACCTAGGATATAGTT; 5′ flankrev: GCTTAGCTAGCCTGAAAATACCTACA). The 3′ flank was a 4925-bp PCR product amplified to continue the genomic homology from a *Nhe*I site at the 3′ end of the 5′ flank. Both forward and reverse primers contained *Not*I sites for cloning into the targeting construct. (3′ flankfor: GCTAAGCGGCCGCAAAGAACACAGTTCAGAAACCTAT; 3′flankrev: ATGGTAGCGGCCGCTACCTAATATCACAAACACA).

Both flanks were cloned into pCR-BluntII-Topo (Invitrogen, Paisley, UK) and sequenced. Clones whose exon sequence was 100% identical to that predicted by the database were chosen to form the targeting construct. Single base changes to intron sequence (compared to the mouse genome sequence, C57Bl/6) were accepted if they occurred in more than one clone. To introduce the required mutation into the 5′ flank the Invitrogen GeneTailor site-directed mutagenesis system was used. The forward mutagenesis primer (GGTGAGGCTGCTCATCCGGAGAtCtGAGTGCTT) changes the coding sequence from proline (CCC) to serine (tCt) and introduces a novel *Bgl*II site for screening for introduction of the mutation. (reverse primer: TCTCCGGATGAGCAGCCTCACCACAACATTT). Plasmids which had been correctly mutagenized were identified by sequencing and digestion with *Bgl*II (pCR-RyR25′P2328S).

**Figure 1:**
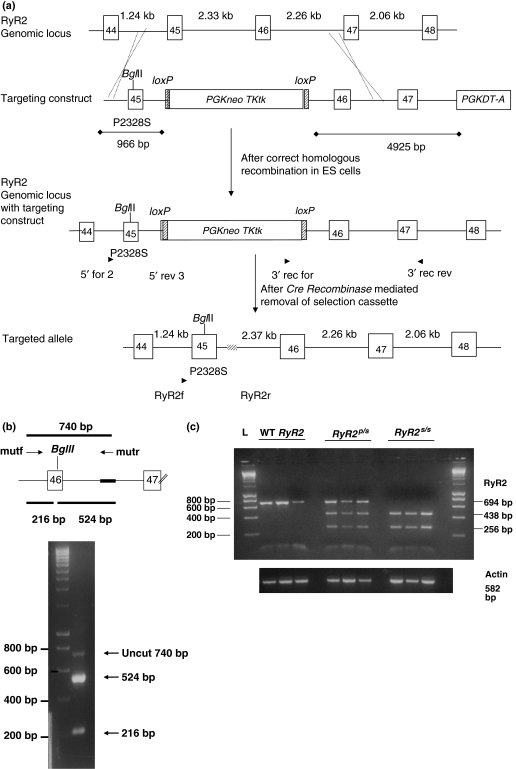
(a) RyR2 P2328S targeting construct (top to bottom; four panels). DNA fragment consisting of introns 44–48 of RyR2 gene (top panel) including the 5.89 kb genomic DNA fragment into which the 966 bp fragment was inserted (second panel). This gave a modified 5′ to 3′ sequence in the embryonic stem (ES) cells (third panel) within the targeted allele (bottom panel). (b) Plasmids correctly mutagenized identified by sequencing and digesting with *Bgl*II to confirm 216, 524 and 740 bp fragments. (c) PCR showing a single 694 bp product in *WT*, three (694, 256 and 438 bp) in *RyR2*^*p/s*^ and two (438 and 256 bp) products in *RyR2*^*s/s*^.

To create the targeting construct, the 3′ flank was cloned into the *Not*I site of pCR-RyR25′P2328SDTA. To reduce non-homologous recombination in the ES cells the PGK promoter-driven Diptheria toxin-A chain gene (from pKO3′/DT-Anew, a kind gift of Dr Michael Snaith) was introduced 3′ of the 3′ flank. The *loxP* flanked *thymidine kinase*/*neomycin* selection cassette (*loxPtkneo*) (from plasmid pNT4) was added between the two flanks to give the final targeting construct, pCR-RyR25′P2328SloxPtkneo3′DTA (Fig. 1a). The targeting construct was checked by sequencing and restriction digestion at each stage of its construction.

Two electroporations of the linearized targeting construct into 129Sv/Ev ES cells were performed from which 132 ES clones were picked. After PCR screening for correct homologous recombination of the 5′ (Hotstar Taq: Qiagen, West Sussex, UK) and 3′ flank (Long Range Taq: Fermentas, York, UK) only one clone was found to be positive. The primer positions for this screening are indicated in Figure 1a. The PCR products of each screen were cloned and sequenced to show that there had been no sequence rearrangements and that the only detectable sequence change was the desired introduction of the codon change. This was confirmed by *Bgl*II digestion. The ES clone was karyotyped to confirm it had the correct number of chromosomes (40).

To remove the *loxPtkneo* selection cassette this clone was electroporated with plasmid expressing *Cre Recombinase* (pPGKnlsCreSV40polyA, a kind gift from Dr Sam Aparicio) and selected in gancyclovir to promote the loss of the *loxPtkneo* selection cassette. Twelve colonies were picked and screened by PCR to check for loss of the selection cassette (Primers RyR2f: GGGGAGTTGGCTGCTGAGAA; RyR2r: CGGGCACACACTCACCACCAA). The PCR product was cloned and sequenced to confirm that it contained the expected RyR2 genomic sequence with 204 bp of vector, including a single *loxP* site, introduced into intron 45 at the *Nhe*I site at the end of the 5′ flank. The karyotype of the new clone was reconfirmed and then this clone was injected into C57Bl/6 blastocysts to produce chimeras. Confirmed germline transmitting chimeras were mated to 129Sv/Ev females. The line was then maintained as a mixture of *RyR2*^*p/s*^ heterozygous matings or *RyR2*^*s/s*^ homozygous matings.

#### Reverse transcription PCR

Total RNA was isolated from mouse ventricle or skeletal muscle using the Qiagen RNeasy fibrous tissue mini kit. The RNA was quantified using a Nanodrop ND-1000 (NanoDrop Technologies, Inc., Wilmington, DE, USA, supplied by LabTech International, East Sussex, UK) and 0.5 μg of RNA was reverse transcribed using random primers and AMV Reverse Transcriptase (both from Promega, Southampton, UK) in the following reaction: RNA was incubated at 70 °C for 10 min, then placed on ice and the following reagents added: 2 μL 0.1 m DTT, 4 μL 5× RT buffer, 1 μL 10 mm dNTPs, 1 μL μg^−1^ Random primers, 0.5 μL RNAguard (Pharmacia, Sandwich, UK), 10 U AMV RT in a total volume of 10 μL. The reaction mix was then incubated at 25 °C for 10 min, 42 °C for 60 min and 70 °C for 15 min. Control reactions in which the RT enzyme was replaced with water were included to exclude amplification from genomic DNA. The resulting cDNA was then diluted to 50 mL and stored at −80 °C. To set up the PCR reaction 2 μL of cDNA was added to 0.5 μL 10 mm dNTPs, 2.5 μL 10× PCR buffer, 0.5 μL 10 pmol μL^−1^ of each primer, 1 U SigmaRedTaq in a total volume of 25 μL. Cycling conditions for PCR: 94 °C 2 min; then 30 repeats 93 °C 30 s, 62 °C 30 s, 72 °C 60 s; followed by 72 °C for 10 min. Primers: RyR2 RT for, GGCGAGTCCAAGGAAATCAC; RyR2 RTRev, CCAGCATGAATCAAGTGCATTTC; mm β actin for, GTTACCAACTGGGACGACATGG, mm β actin rev, CCATACCCAAGAAGGAAGGCTG. The PCR products were digested by addition of *Bgl*II to the PCR mix and incubation at 37 °C for 2 h. The digest was analysed by electrophoresis on a 2% agarose gel.

All mice used were originally supplied by Harlan (Oxon, UK) and were housed at room temperature and subjected to 12-h light/dark cycles. They were fed sterile rodent chow and had free access to water at all times. Mice used were wild-type (*WT*), heterozygote (*RyR2*^*p/s*^) and homozygote (*RyR2*^*s/s*^) all aged between 3 and 6 months. Experimental mice were bred from a 129 genetic background, which, along with C57 mice, are less susceptible to PES-induced arrhythmias than FBV or Black Swiss animals (Maguire *et al.* 2003). All mice used for subsequent experimental work were killed by cervical dislocation [Schedule 1: UK Animal (Scientific Procedures) Act 1986].

### Measurements of cell [Ca^2+^]

Single-cell experiments used mouse ventricular myocytes isolated adapting an established enzymatic digestion as described previously (Harding *et al.* 1992). Following killing, hearts were excised rapidly and cannulated before being connected to a Langendorff perfusion system. The heart was then perfused for 1.5 min at 3 mL min^−1^ with perfusion buffer containing (mm), 120 NaCl, 5 MgSO_4_, 5.4 KCl, 5 sodium pyruvate, 10 Hepes, 5 glucose, 20 taurine, 5 nitrilotriacetic acid (NTA; Sigma-Aldrich, Poole, UK), and 1–2 μm Ca^2+^, pH 7.0 at 37 °C. Subsequently the heart was perfused for 25–28 min at 3 mL min^−1^ with digestion buffer containing (mm), 120 NaCl, 5 MgSO_4_, 5.4 KCl, 5 sodium pyruvate, 10 Hepes, 5 glucose, 20 taurine, to which was added collagenase type II (final concentration 1 mg mL^−1^; Worthington Biochemical, Lakewood, NJ, USA), hyaluronidase (final concentration 1 mg mL^−1^; Sigma-Aldrich) and 13 μm Ca^2+^ and set at pH 6.8 at a temperature of 37 °C. The pale and swollen heart was then removed and cut below the atria. The digested tissue was teased gently with fine forceps and the dissociated cells were transferred to a conical tube containing 12.5 mL digestion buffer with 50 mg bovine serum albumin (BSA) to inactivate digestion. The resulting myocytes were subject to further trituration using sterile plastic transfer pipettes, and allowed to sediment by gravity for 10 min.

The cell suspension was then centrifuged for 1.5 min at 200 rpm and the pellets re-suspended twice in wash buffer containing (mm): 119 NaCl, 4.2 KCl, 0.94 MgSO_4_, 1.2 KH_2_PO_4_, 20 Hepes, 11.5 glucose, 20 taurine and 1 mg mL^−1^ BSA, and bubbled with 95% O_2_ + 5% CO_2_, pH 7.4, to remove all traces of NTA, collagenase and hyaluronidase. CaCl_2_ was then cautiously re-introduced by stepwise additions of CaCl_2_ to reach an approximate concentration of 1.25 mm by centrifuging the preparation at each Ca^2+^ reloading stage to remove dead or swollen cells. During this process, myocytes were examined periodically under the microscope to confirm a good yield of rod-shaped myocytes (better than 60%) before continuing the experiments. Cells were then transferred into a conical tube (BD Biosciences, Oxford, UK) and incubated at room temperature. The isolated myocytes were then placed in a control Hepes-buffered Krebs-Henseleit (KH) solution in a Perspex chamber 10 × 4 × 6.25 mm (length × width × depth): the myocytes spontaneously attached to the glass cover slip (4 × 4 cm, grade 1 cover slip; (Merck, Herts, UK) forming the floor of the chamber. The myocytes were stimulated to contract using two in-built platinum field electrodes running the length of the chamber through which the periodic field stimulation was applied using a home-built square-wave stimulator (R. Montgomery, Imperial College, London, UK). This applied successive 40–60 V steps of 2.2 ms duration, immediately followed by a step of similar duration and amplitude but opposite polarity at a pacing frequency of 0.5 Hz.

During this stimulation cells were loaded with the acetoxymethyl (AM) ester of the Ca^2+^-sensitive dye Fluo-3 (F-14242; Invitrogen, Paisley, UK; 50 μg vial diluted in 30 μL pluronic to give a stock concentration of 1.476 mm) by incubating the cells in a bath of volume 500 μL containing KH buffer (with 1.25 mm Ca^2+^) with 2 μL of Fluo-3 AM solution for 15 min at room temperature in the dark. The bath with the myocytes was then transferred onto the stage of a Zeiss LSM-510 laser scanning confocal microscope system (Carl Zeiss, Welwyn Garden City, UK) with a ×20 air objective (NA 0.5; confocal aperture 1000 μm, slice thickness <42.4 μm) on a Zeiss Axiovert 100 M inverted microscope. The fluorescence (*F*) signals resulting from Ca^2+^ binding to fluo-3 were excited by a 488-nm argon laser and detected between wavelengths of 505–550 nm. Images were analysed using an in-house custom-written program. Series of 500 frames sampled at 98 ms per frame (128 × 128 pixels per frame) were used initially to monitor fluorescence changes over time. The appropriateness of the chosen frame capture rate was corroborated in some experiments by confirming similar results following use of the faster line-scan mode with a sampling rate of 960 μs per frame. Fluorescence signals were quantified within three successive regions of interest (ROI) placed at 7 μm intervals along the long axis of each myocyte. Each ROI was circular, with an area of ∼50 μm^2^. Ca^2+^ transients obtained in response to the regular pacing stimuli were quantified in terms of their peak values that were normalized to the baseline fluorescence level *F*_0_, to give peak *F/F*_0_ values following each response to each stimulus. All values were calculated from three ROIs for each myocyte and the mean peak *F*/*F*_0_ values as well as the baseline diastolic values were calculated. All traces had stable baselines confirming consistent laser intensities and detector gains. The results were expressed as mean ± SEM and compared using anova (spss software, SPSS, Chicago, IL, USA).

### Whole heart electrophysiological experiments

The electrophysiological experiments on arrhythmogenesis at the whole heart level used a Langendorff-perfused preparation adapted for the murine model (Papadatos *et al.* 2002, Balasubramaniam *et al.* 2003). The contracting heart was stimulated with platinum-stimulating electrodes applied to the epicardial surface, usually 15 min at 10 Hz, and was allowed to reach its physiological steady state. The heart was paced from its right ventricle at three times its threshold level (between 3 and 5 V) using square-wave stimuli of 2 ms duration (Grass S48 stimulator; Grass Telefactor (UK), Slough, UK). Monophasic action potentials (MAPs) were recorded from the epicardial basal surface of the left ventricle using a MAP electrode (Linton Instruments, Harvard Apparatus, Edenbridge, UK).

Hearts were subject to two stimulation protocols. First, they were regularly paced at 6, 8, 10 and 12 Hz for periods of up to 30 min to assess for tendencies to spontaneous arrhythmogenesis. Secondly, programmed electrical stimulation (PES) of the heart was then carried out using a variation of an existing clinical technique (Saumarez & Grace 2000, Saumarez *et al.* 2003). This applied stimulation sequences that each consisted of a drive train of pacing stimuli (S1) applied with a 200 ms cycle length with an extra stimulus (S2) inserted every eighth beat. The coupling interval (S1S2) was successively reduced by 1 ms with each cycle until the ventricular effective refractory period (VERP) was reached. The MAP signals were amplified, filtered (band-pass filter 30 Hz to 1 kHz; Gould-Nicolet Technologies, Ilford, Essex, UK) and digitized using an analog-to-digital converter (Model 1401plus; Cambridge Electronic Design, Cambridge, UK).

The MAP signals were analysed with Spike II software (Cambridge Electronic Design) and MAP traces were selected according to previously documented criteria requiring stable baselines with rapid upstrokes and consistent amplitudes. The action potential durations (APD) at 30, 50, 70 and 90% repolarization (APD_30,_ APD_50_, APD_70_ and APD_90_) were fully identified and tabulated in Excel (Microsoft, Redmond, WA, USA). Results were all expressed as mean ± SEM comparing different groups of experimental animals using anova (spss software).

Isoproterenol was dissolved initially in distilled water to make a stock of 1 mm and the final drug concentration was achieved by dilution with the control KH buffer. The stocks were stored at −20 °C and made fresh on the day of each experiment. These agents were obtained from Sigma-Aldrich.

## Results

### RyR2^P2328S^ mice

The three injections of embryonic stem cells into C57B/6 blastocysts resulted in a total of eight male chimeras. Each of these males was mated with Ca 57Bl/6 and a 129Sv/Ev female. Three of the chimeras showed germ line transmission of the knock-in allele, two produced no litters and two produced litters but did not show germ line transmission. Genotyping of pups was performed using primers RyR2f and RyR2r (Fig. 1a). These span the vector sequence left in intron 46. A second PCR can be used to confirm the presence of the *RyR2*^*P2328S*^ allele. The reverse primer binds to vector sequence left in intron 45 and the primers span the introduced mutation to produce a 740-bp product which can be cut by *Bgl*II to produce two, 524 and 216 bp, products (Fig. 1b). Pups were genotyped at 3–4 weeks of age. Results of genotyping 404 mice gave 98 *WT*, 189 *RyR2*^*p/s*^ and 117 *RyR2*^*s/s*^. This is not statistically different from the expected numbers of 101, 202 and 101. The oldest mice in the colony were 18 months of age. There has been no significant additional mortality of either *RyR2*^*p/s*^ or *RyR2*^*s/s*^ compared with *WT*.

To check that both alleles produced mRNA, RT-PCR was performed on total RNA isolated from mouse ventricle tissue. Figure 1c shows the results of RT-PCR after the product had been digested by *Bgl*II and the digest run on a 2% agarose gel. For each genotype the RT-PCR reaction was run in triplicate. RNA from WT mice produced a single product at 694 bp. RNA from the heterozygous *RyR2*^*p/s*^ mice produced three products, uncut WT allele and digested *RyR2*^*P2328S*^ allele. RNA from the homozygous *RyR2*^*s/s*^ mice produced only two products (438 and 256 bp) showing that the RT-PCR product is derived from the *RyR2*^*P2328S*^ allele only. The β-actin gene was amplified in all samples as a positive control. PCR reactions using the −RT cDNA were also included as negative controls. No amplification was seen in these reactions from either the RyR2 or β-actin primers (data not shown). To show that the RyR2 primers were not amplifying from RyR1-derived cDNA, skeletal muscle-derived cDNA was used as a template. No amplification from the RyR2 primers was seen in these reactions, but the β-actin primers were able to amplify the expected product from the same samples (data not shown).

### The presence of the P2328S allele alters Ca^2+^ homeostasis

**Figure 2:**
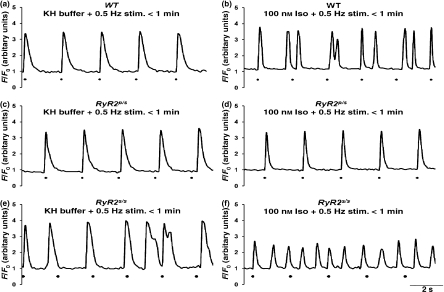
Ca^2+^ peaks in regularly stimulated (0.5 Hz) Fluo-3-loaded *WT* (a, b), *RyR2*^*p/s*^ (c, d) and *RyR2*^*s/s*^ (e, f) before (a, c, e) and following (b, d, f) introduction of 100 nm isoproterenol (Iso). Each trace depicts the initial 10 s obtained from a 50-s sequence immediately following Fluo-3 loading.

Differences in cellular Ca^2+^ homeostasis that might relate to the presence of the *P2328S* allele were first investigated through changes in the amplitude and pattern of Ca^2+^ transients in response to regular stimulation (0.5 Hz) in isolated Fluo-3-AM loaded *WT* (Fig. 2a,b), *RyR2*^*p/s*^ (Fig. 2c,d) and *RyR2*^*s/s*^ (Fig. 2e,f) myocytes. These were studied in KH buffer in both the absence and presence of isoproterenol at a concentration (100 mm) at which it would be possible to assess the extent to which the *RyR2*^*p/s*^ or *RyR2*^*s/s*^ mutation might either increase or decrease the incidence of ectopic or subsidiary Ca^2+^ events. In all three groups, the traces showed stable baseline *F/F*_0_ and reproducible responses to regular stimulation with minimal evidence of fluorophor bleaching over the sampling periods. Thus the average values of the diastolic baseline *F/F*_0_ were statistically indistinguishable (*P* >> 5%) between all three groups whether in the presence and absence of isoproterenol. Similar stability conditions applied to the corresponding peak *F*/*F*_0_ amplitudes. We further quantified the characteristics of such peak *F*/*F*_0_ (Table 1) obtained from Ca^2+^ transients of the kind illustrated in Figure 2. In the absence of isoproterenol, Ca^2+^ signals observed immediately after the commencement of stimulation in *WT* (Fig. 2a) and *RyR2*^*p/s*^ myocytes (Fig. 2c) consisted of a series of responses each directly following the individual stimuli. *WT* (Fig. 2a) and *RyR2*^*p/s*^ myocytes (Fig. 2c) similarly did not show either subsidiary events during the recovery time courses of the evoked transients or spontaneous ectopic peaks in the intervals between these regular responses to the imposed stimuli. In contrast, the *RyR2*^*s/s*^ myocytes showed an irregular pattern of responses that included subsidiary events (4.65 ± 1.46 events counted over a standard sampling period of 50 s; *n*=36 cells, three mice) (Fig. 2e). After the addition of isoproterenol, *WT* myocytes showed both ectopic peaks (8.00 ± 2.99; *n*=6 cells, two mice) and subsidiary events (9.00 ± 1.34, *n*=6 cells, two mice) (Fig. 2b), and *RyR2*^*s/s*^ myocytes showed an increased incidence of ectopic peaks (15.83 ± 6.76) compared to the correspondingly isoproterenol treated *WT*, but no subsidiary events. *RyR2*^*p/s*^ myocytes showed neither ectopic peaks nor subsidiary events (Fig. 2 and Table 1). In contrast, Table 1 indicates that the peak *F*/*F*_0_ of Ca^2+^ transients directly evoked by each of the individual stimuli were statistically indistinguishable on independently weighted anova in *WT*, *RyR2*^*p/s*^, *RyR2*^*s/s*^ regardless of whether or not isoproterenol was present with the exception of a reduced peak *F*/*F*_0_ in isoproterenol-treated *RyR2*^*s/s*^, as expected for a depletion of SR Ca similarly produced by isoproterenol and caffeine (Venetucci *et al.* 2007).

**Table 1:** Comparison of the characteristics of Ca^2+^ signals in regularly stimulated *WT*, *RyR2*^*p/s*^ and *RyR2*^*s/s*^ cardiac myocytes

	*WT*	*RyR2*^*p/s*^	*RyR2*^*s/s*^
Mean peak *F*/*F*_0_ ± SEM			
Krebs buffer (*n*)	3.04 ± 0.22 (6)†	3.15 ± 0.06 (12)†	3.34 ± 0.08 (36)†***
Isoproterenol (*n*)	2.88 ± 0.25 (6)†	3.16 ± 0.04 (12)†	1.59 ± 0.10 (18)***
Mean numbers of ectopic peaks			
Krebs buffer (*n*)	0 ± 0.0 (6)***	0 ± 0.0 (12)	0.79 ± 0.56 (36)**
Isoproterenol (*n*)	8.00 ± 2.99 (6)***	0 ± 0.0 (12)	15.83 ± 6.76 (18)**
Mean numbers of evoked responses with subsidiary events			
Krebs buffer (*n*)	0 ± 0.0 (6)***	0 ± 0.0 (12)	4.65 ± 1.46 (36)*
Isoproterenol (*n*)	9.00 ± 1.34 (6)***	0 ± 0.0 (12)	0 ± 0.0 (18)*

*WT*, wild-type; *RyR2*^*p/s*^, heterozygote; *RyR2*^*s/s*^, homozygote.

Symbols indicate values subject to one-way anova of findings obtained between designated groups, or in each group before and after introduction of isoproterenol with the following results:

† *P* = ns

* *P*<0.05

** *P*<0.01

*** *P*<0.001.

**Figure 3:**
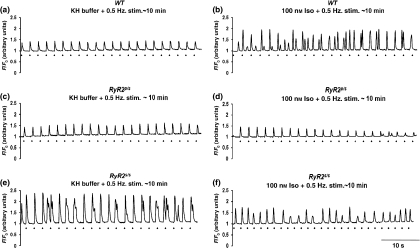
Ca^2+^ peaks in regularly stimulated (0.5 Hz) Fluo-3-loaded *WT* (a, b), *RyR2*^*p/s*^ (c, d) and *RyR2*^*s/s*^ (e, f) before (a, c, e) and following (b, d, f) introduction of 100 nm isoproterenol (Iso). Each trace depicts a 50-s sequence obtained 10 min following commencement of stimulation following Fluo-3 loading.

**Figure 4:**
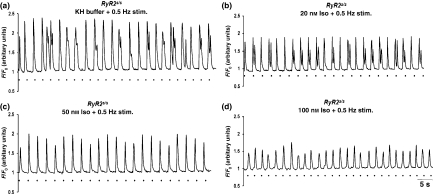
Ca^2+^ transients in regularly stimulated (0.5 Hz) Fluo-3-loaded *RyR2*^*s/s*^ myocytes before (a) and following introduction of 20 nm (b), 50 nm (c) and 100 nm (d) isoproterenol (Iso). Each trace was obtained from a 50-s sequence immediately following fluo-3 loading.

Further observations were made in cells following continual stimulation extending over ∼10 min. Resumption of scanning then confirmed a similar appearance of subsidiary events in *RyR*^*s/s*^ but not *WT* or *RyR2*^*p/s*^ myocytes (Fig. 3a,c,e). Addition of isoproterenol then similarly resulted in the appearance of both ectopic peaks and subsidiary events in *WT* (Fig. 3b) and their continued absence in *RyR2*^*p/s*^ myocytes (Fig. 3d). Such results agreed with the findings made immediately following the onset of stimulation (Fig. 2) despite the now reduced peak *F*/*F*_0_ values likely reflecting progressive SR Ca^2+^ depletion by such prolonged stimulation. *RyR2*^*s/s*^ myocytes showed irregular patterns of release events (Fig. 3f). These were further studied (Fig. 4) under conditions of exposure to 0 (*n*=7), 20 (*n*=4), 50 (*n*=4) and 100 nm (*n*=5) isoproterenol respectively: this gave progressive (and significant) reductions in peak *F/F*_0_ from 4.045 ± 1.021 to 3.846 ± 0.068 (*n*=5), 1.63 ± 0.0048 (*n*=5) and 1.470 ± 0.058 (*n*=5; *P*=0.03 on independent weighted analysis), in which a reduction in the frequency of subsidiary peaks from 16.33 ± 2.08 to 6.57 ± 1.72 and 5.8 ± 4.82, following a series of 22 stimulations in 20 and 50 nm isoproterenol, respectively, was replaced by the irregular pattern of release events at 100 nm isoproterenol (Fig. 4d).

**Figure 5:**
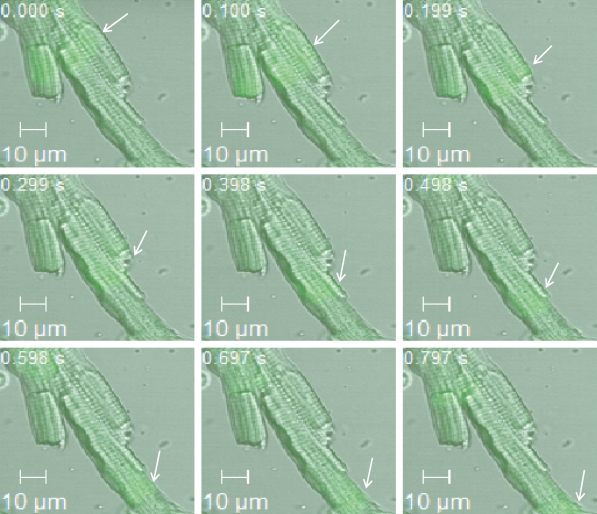
A series of confocal microscope images, obtained from complete galleries showing similar patterns, depicting typical Ca^2+^ waves in a single ventricular *RyR2*^*s/s*^ myocyte loaded with Fluo-3 in the presence of Krebs-Henseleit buffer with 1.25 mm Ca^2+^ following perfusion with 100 nm isoproterenol. The arrows highlight the path taken by a typical Ca^2+^ wave.

*RyR2*^*s/s*^ cells demonstrated Ca^2+^ waves hitherto associated with arrhythmogenic properties in caffeine-treated murine myocytes (Balasubramaniam *et al.* 2005), following the addition of isoproterenol. As described above, Ca^2+^ signals, synchronized to the applied stimuli, were observed in the presence of KH buffer with 1.25 mm Ca^2+^ for the *WT* and *RyR2*^*p/s*^ myocytes before and following addition of 100 nm isoproterenol. However, addition of 100 nm isoproterenol resulted in an appearance of propagated Ca^2+^ waves along the longitudinal axis of the cell in the *RyR2*^*s/s*^ (Fig. 5). The arrows highlight the path taken by the Ca^2+^ wave, starting from the upper end of the cell and moving along its length. Similar results were observed in *n*=5 cells.

### Action potentials from hearts containing the P2328S allele during regular pacing

Experiments on isolated Langendorff-perfused whole hearts compared electrophysiological properties of *WT*, *RyR2*^*p/s*^ and *RyR2*^*s/s*^ hearts, exploring for arrhythmogenic tendency both before and following addition of 100 nm isoproterenol in each heart. Mice were aged between 3 and 6 months (*WT*: one female, two males; *RyR2*^*p/s*^: four females, four males; and *RyR2*^*s/s*^: one female, six males). The experiments used both regular pacing and PES procedures, which imposed an S2 extra stimulus following eight-beat (S1) conditioning sequences at S1S2 intervals progressively shortened with each stimulus cycle. Experiments used pacing (S1) frequencies of 6, 8, 10 and 12 Hz respectively. *WT*, *RyR2*^*p/s*^ and *RyR2*^s/s^ hearts paced at 10 Hz showed statistically indistinguishable VERPs of 63 ± 11.2 (*n*=3), 56.3 ± 8.4 (*n*=3) and 66.5 ± 18.5 ms (*n* = 3 hearts) respectively.

**Figure 6:**
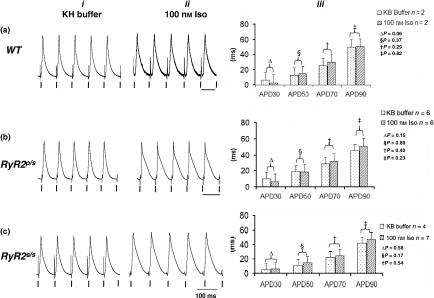
Monophasic action potential waveforms of whole isolated perfused hearts from *WT* (a), *RyR2*^*p/s*^ (b) and *RyR2*^*s/s*^ (c), before (*i*) and after (*ii*) the addition of 100 nm isoproterenol (Iso); (*iii*) quantifies traces of this kind and plots action potential duration (APD) values at 30, 50, 70, and 90%, APD_30, 50, 70, 90._ The results of the paired *t*-test for APD values obtained in the absence and presence of isoproterenol in each genotype are shown. These did not show any significant differences for all three genotypes.

The experiments first examined MAP waveforms in hearts regularly paced at 10 Hz for at least 30 min in KH buffer before and after addition of isoproterenol. They assessed for the presence or absence of changes in APD of the kind reported for LQTS mice models on recent occasions (Head *et al.*, 2005; Stokoe *et al.*, 2007a,b; Thomas *et al.*, 2007a,b). APD values measured at 30, 50, 70 and 90% recovery (APD_30, 50, 70, 90_) from regularly paced *WT* (*n*=3 hearts), *RyR2*^*p/s*^ (*n*=7 hearts) and *RyR2*^*s/s*^ (*n*=7 hearts) before and 20 min after addition of isoproterenol indicated uniform MAPs (Fig. 6a*i,ii*, b*i,ii* and c*i,ii* respectively), with no significant differences in all these APD durations between *WT* (Fig. 6a*iii*), *RyR2*^*p/s*^ (Fig. 6b*iii*) and *RyR2*^*s/s*^ hearts (Fig. 6c*iii*). Such findings parallel reports on a previously described R4496C mutant where QT intervals, of WT and the mutant mice did not present significant differences whether before or after adrenergic stimulation (Cerrone *et al.* 2005). These data therefore parallel findings that CPVT carriers of the *RyR2*-*P2328S* mutation (Paavola *et al.* 2007) showed increases in endocardial MAP durations considerably smaller than the clinical QTc prolongations thought to account for the LQTS phenotype (Keating & Sanguinetti, 2001; Scoote & Williams, 2002; Laurita & Katra, 2005; Priori & Napolitano, 2005).

### Arrhythmic properties of hearts containing the P2328S allele during regular pacing and programmed electrical stimulation at varying frequencies

**Figure 7:**
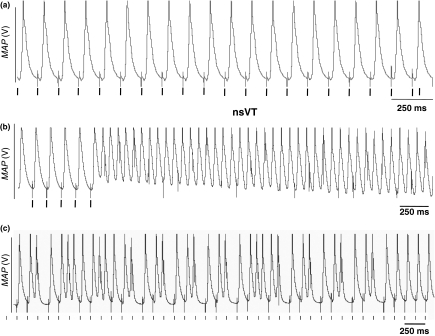
Monophasic action potential waveforms of whole isolated perfused hearts in Krebs-Henseleit buffer from *RyR2*^*s/s*^ (a). Of the three groups studied, *RyR2*^*s/s*^ hearts showed the highest incidence of ventricular tachycardias (b) which could be either sustained (>30 s) or non-sustained (<30 s) and early after depolarizations (EADs) (asterisks) (c). Results shown for hearts subject to regular pacing at 8 Hz.

The presence or absence of an arrhythmogenic phenotype was then investigated in the *WT*, *RyR2*^*p/s*^ and *RyR2*^*s/s*^ hearts subject to both regular pacing and PES. VT was identified as a series of regular electrical deflections recurring at high frequencies independent of any pacing spikes that were present. Arrhythmias lasting >30 s were defined as being sustained (sVT) and those lasting <30 s as being non-sustained (nsVT). On these criteria: (1) *regularly paced WT hearts showed no arrhythmic incidents at all frequencies* (6, 8, 10 and 12 Hz) explored whether isoproterenol was present (*n*=3) or absent (*n*=4 hearts) (data not shown). (2) *The RyR2*^*p/s*^*hearts showed relatively few arrhythmogenic phenomena, when regularly paced, in the absence of isoproterenol.* Thus, of seven hearts, only one then showed an episode of sVT at a pacing rate of 12 Hz. However, addition of isoproterenol (100 nm) enhanced arrhythmic tendency with 1, 1 and 0 episodes of nsVT at pacing frequencies of 6, 8 and 10 Hz and 0, 1, 0 and 1 episodes of sVT at a pacing frequency of 6, 8, 10 and 12 Hz respectively (*n*=5 hearts). (3) *RyR2*^*s/s*^*hearts showed noticeably greater arrhythmic tendencies even prior to addition of isoproterenol* (Fig. 7). Two hearts then showed episodes of nsVT at 8 and 10 Hz (Fig. 7b), and two further hearts showed episodes of sVT at 10 Hz (*n*=11 hearts). (4) *Addition of isoproterenol increased this arrhythmic tendency.* Thus, its introduction resulted in (*n*=5 hearts) 2, 1, 0 and 0 episodes of sVT at pacing frequencies of 6, 8, 10 and 12 Hz respectively. Finally, spontaneous after-depolarizations, previously implicated in the triggering of arrhythmias, were observed in hearts paced at both 8 and 10 Hz (*n*=2) (Fig. 7c).

**Figure 8:**
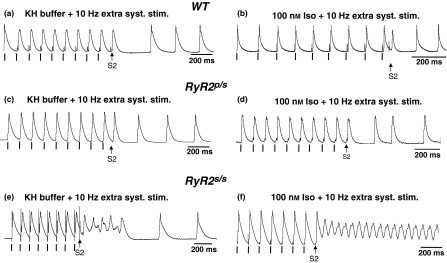
Monophasic action potentials (APs) recorded from the epicardium of *WT* (a, b), *RyR2*^*p/s*^ (c, d) and *RyR2*^*s/s*^ hearts (e, f) before (a, c, e) and after (b, d, f) the addition of 100 nm isoproterenol (Iso). All experiments were performed during programmed electrical stimulation (PES) at 10 Hz; the arrows indicate S2 extra-stimuli. Of the events observed the examples illustrated of the *WT* (a) and *RyR2*^*p/s*^ (c) isolated perfused hearts showed persistent regular rhythm with no arrhythmogenic events at 10 Hz during the PES procedures. Five of seven *RyR2*^*p/s*^ hearts (1, 1, 2, 3 episodes at 8, 8, 10 and 12 Hz respectively) showed nsVT. In four of five *RyR2*^*p/s*^ hearts studied in the presence of 100 nm isoproterenol, 0, 3, 3, and 1 hearts showed nsVT at pacing frequencies of 6, 8, 10 and 12 Hz, respectively, and one heart showed sVT both at 10 and 12 Hz. The *RyR2*^*s/s*^ hearts showed 3, 3, 3, and 5 episodes of nsVT and 0, 1, 3 and 1 episodes of sVT at pacing rates of 6, 8, 10 and 12 Hz respectively (*n*=11 hearts). Addition of 100 nm isoproterenol (*n*=5) resulted in a high incidence (2, 3, 3 and 4 episodes) of sVT. In the example shown, the S2 extra-stimuli initiated an episode of non-sustained VT lasting for less that 30 s in KB buffer alone (e). Sustained VT lasting for more than 30 s was often observed following addition of 100 nm isoproterenol in the same heart during PES at 10 Hz (f). Scale bar 200 ms.

The subsequent electrophysiological experiments explored for arrhythmogenic substrate as reflected in initiation of VT by extrasystolic (S2) stimulations applied during PES procedures (Fig. 8). This gave results that correlated well with the pacing studies. They demonstrated that (5) *WT hearts were not arrhythmogenic at all pacing frequencies*, whether before (*n*=4, Fig. 8a) or after (*n*=3, Fig. 8b) introduction of isoproterenol. In contrast, (6) *both RyR2*^*p/s*^*and RyR2*^*s/s*^*hearts showed an arrhythmogenicity exacerbated by isoproterenol.* Thus, five of seven *RyR2*^*p/s*^ hearts (Fig. 8c) showed nsVT at all applied pacing frequencies. Isoproterenol (100 nm) increased this incidence in four of five hearts. The *RyR2*^*s/s*^ hearts showed not only higher incidence of nsVT, but also an occurrence of sVT (*n*=11 hearts, Fig. 8e). Addition of 100 nm isoproterenol resulted in full sVT (*n*=5) (Fig. 8f). Taken together, these results suggest that the *RyR2*-*P2328S* mutation results in increased tendencies to nsVT as well as sVT in mice. This is common with features shown by CPVT patients with *RyR2* mutations (Swan *et al.* 1999, Laitinen *et al.* 2001, Priori *et al.* 2001, 2002).

### RyR2^s/s^ hearts additionally show arrhythmic behaviour during intrinsic activity

**Figure 9:**
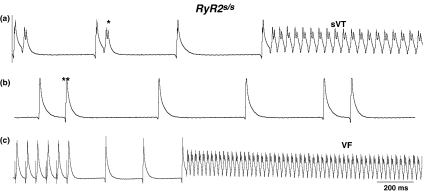
Monophasic action potentials (APs) recorded from the epicardium of intrinsically paced initially non-arrhythmogenic *RyR2*^*s/s*^ hearts. Experiments were performed in the absence (a, b) and presence of electrical stimulation prior to (c). Of the spontaneous events observed both (a): early-after depolarizations (EADs) (*) that produced episodes of sustained monomorphic VT (sVT) when stimulation resumed, and (b): coupled beats (**) were observed in six of 11 hearts. (c) Persistent ventricular fibrillation (VF) occurring following the cessation of regular S1 pacing after two intrinsic MAPs.

Spontaneous arrhythmogenic phenomena were only observed in intrinsically paced *RyR2*^*s/s*^ and not in *WT* (*n*=3) or *RyR2*^*p/s*^ (*n*=7). Six of 11 *RyR2*^*s/s*^ hearts showed early after depolarizations (EADs) that intercepted the recovery phase of their preceding action potentials (Fig. 9a), and 11 of 11 showed coupled beats consisting of pairs of full action potentials (Fig. 9b). There was one example of nsVT following the coupled beats. When stimulation was ceased and re-introduced at a pacing rate of 10 Hz, it resulted in an episode of ventricular fibrillation (Fig. 9c). These findings are consistent with reports in which CPVT patients exhibited episodes of polymorphic VT leading to SCD while asleep or at rest (Allouis *et al.* 2005, d’Amati *et al.* 2005, Postma *et al.* 2005).

## Discussion

Catecholaminergic polymorphic ventricular tachycardia (CPVT) associated with SCD (Wehrens & Marks 2003, Francis *et al.* 2005, George *et al.* 2007), has been linked to mutations in the gene encoding the ryanodine receptor (RyR2)-sarcoplasmic reticular Ca^2+^ release channel (Swan *et al.* 1999, Priori *et al.* 2001). The mutations concerned are primarily clustered in three regions of the gene namely, the N-terminal region (176–433), central domain (2246–2504) and the C-terminal region (4104–4653) (Wehrens & Marks 2003).

In contrast to murine models directed to the C and N-terminal regions, there have been no such models involving the important central region (Cerrone *et al.* 2005, Kannankeril *et al.* 2006). This study reports the generation of mice with a knock-in mutation in the central domain of the *RYR2* gene (P2328S). The variant was first identified, in common with carriers for the Q4201R and V4653F in three unrelated Finnish families. The P2328S mutation is located in the large central cytoplasmic domain of RyR2, near the binding site of the regulatory protein FKBP12.6 (George *et al.* 2007); the Q4201R and V4653F mutations occur in the carboxy-terminal region of the receptor associated with the pore region (Tunwell *et al.* 1996). All the human carriers showed mortality rates of ≈33% between the ages of 10 and 35 years and threshold heart rates of ∼130 beats min^−1^ for the triggering of ventricular arrhythmogenesis during exercise despite an absence of structural abnormalities or heart failure (Laitinen *et al.* 2001). Recent clinical studies of carriers for the P2328S, as well as the Q4201R and V4653F, mutations reported runs of polymorphic tachycardia during exercise with a mean age of onset of symptoms of 34 ± 16 years. Furthermore, endocardial MAP recordings at the right ventricular septum from patients with one of the three *RyR2* mutations showed delayed afterdepolarizations (DADs) in three of 15 CPVT patients. These occasionally coincided with premature ventricular complexes both before and during adrenaline infusion that were never observed in control subjects (Paavola *et al.* 2007).

There has been extensive discussion about the association between mutations in the *RyR2*, including that of P2328S, and the CPVT phenotypes, particularly concerning possible roles of FKBP12.6-RyR2 binding (Brillantes *et al.*, 1994; Masumiya *et al.* 2003, Zissimopoulos & Lai 2005, Thomas *et al.* 2006). Three *RyR2* CPVT mutations (S2246L, R2474S and R4497C) have been associated with low channel activity in the absence of PKA at a wide range of cytosolic [Ca^2+^] and enhanced sensitivity to PKA activation increasing open RyR2 probabilities (Wehrens *et al.* 2003). Similarly, investigations for arrhythmic mechanisms following adrenergic stimulation in HEK293 cells expressing P2328S (and Q4201R and V4653F) mutations suggested a gain-of-function defect with leaky Ca^2+^ release channels showing significant rightward shift in half-maximal inhibitory Mg^2+^ concentration and decreased FKBP12.6 binding (Lehnart *et al.* 2004, Wehrens *et al.* 2004, Paavola *et al.* 2007).

However, other studies have reported that mutations in the RyR2 central region variously showed increased (N2386I and Y2392C) and decreased (R2427S, S2246L and P2328S) FKBP12.6 binding affinity (Tiso *et al.* 2002 and Wehrens *et al.* 2003 respectively), although a decreased affinity with C-terminal mutations (Q4201R, R4496C and V4653F) (Wehrens *et al.* 2003). S2808D-myotubes did not show spontaneous Ca^2+^ oscillations at any concentration of FKBP12.6 (Goonasekera *et al.*, 2005). Fluorescence resonance energy transfer investigations of the functional impact of C-terminal and central domain *RyR2* mutations suggested effects on RyR2 channel activity independent of FKBP12.6 binding and PKA phosphorylation (George *et al.*, 2004 and reviewed in George *et al.* 2003, 2006, Jiang *et al.* 2004, Liu *et al.* 2006). Such discrepancies led to alternative suggestions implicating elevations of SR store Ca^2+^ content to a threshold level producing spontaneous SOICR with unaltered FKBP12.6-RyR2 interaction in CPVT. SOICR occurred in HEK293 cells expressing *WT* RyR2 exposed to elevated extracellular [Ca^2+^]_0_ (Diaz *et al.* 1997, Jiang *et al.* 2004). It also occurred in cells expressing RyR2 containing the N4104K, R4496C and N4895D mutations, as well as further *RyR2* mutations from central (S2246L and R2474S), C- (Q4201R and I4867M) and N-terminal (R176Q (T2504M) and L433P) regions, in the form of increased frequencies of Ca^2+^ oscillations and decreased SR Ca content. Single mutant RyR2 channels showed increased activation sensitivities with a luminal Ca^2+^ of 300 μm and increased basal [^3^H] ryanodine binding (see also Jiang *et al.* 2005). Such findings were corroborated using the first *RyR2-R4496C* (*+/−*) knock-in mouse model equivalent to the R4497C mutation identified in CPVT patients. Five of 14 such mice showed either nsVT or sVT only with exercise stress testing followed by adrenaline administration. Four of eight of a second group developed arrhythmias following adrenaline and caffeine administration, as did four of five pre-treated with K201 (Cerrone *et al.* 2005). Recent optical mapping studies further characterized these arrhythmogenic characteristics (Cerrone *et al.* 2007). Subsequent analyses demonstrated that pacing protocols of increasing frequencies induced DADs in *RyR2-R4496C* (*+*/−) cardiomyocytes but not in the *WT*. Superfusion with isoproterenol induced both DADs and triggered activity in the *RyR2-R4496C* (+/−) but not in the *WT*. Finally, the heterozygote showed slightly increased RyR2-FKBP12.6 interaction compared with *WT* that was unchanged in the presence or absence of caffeine and adrenaline (Liu *et al.* 2006).

Our data provide complementary mouse models using the *P2328S* mutation, at a central site remote from the luminal C-terminal, R4496C site. We have for the first time compared all three genotypes (*WT*, *RyR2*^*p/s*^ and *RyR2*^*s/s*^) at both the single-cell and whole heart level. The presence of *P2328S* allele appears to impart an arrhythmogenic tendency, greater in both *RyR2*^*p/s*^ and *RyR2*^*s/s*^ compared with *WT*. We report successively greater effects on Ca^2+^ transients, arrhythmogenicity, the effect of β-adrenergic stimulation, and the presence and absence of afterpotentials in the order *WT*< *RyR2*^*p/s*^ < *RyR2*^*s/*s^, adding important observations concerning the effects of gene dosage. Thus, there was an increased amplitude of Ca^2+^ transients evoked by regular stimulation in isolated ventricular myocytes with ectopic Ca^2+^ peaks occurring specifically in the *RyR2*^*s/s*^ myocytes as was observed following administration of isoproterenol to the *WT*. Isoproterenol reduced such transients in *RyR2*^*s/s*^, suggesting Ca^2+^ store depletion and an appearance of propagated Ca^2+^ waves associated with arrhythmogenic tendencies on earlier occasions (Venetucci *et al.* 2007). In contrast, MAPs from isolated Langendorff-perfused *WT*, *RyR2*^*p/s*^ and *RyR2*^*s/s*^ hearts had similar waveforms and refractory periods, whether in the presence or absence of isoproterenol. Nevertheless, both regular pacing through a range of frequencies and extrasystolic stimuli demonstrated a progressively increasing tendency to the development of non-sustained, then sustained, arrhythmogenesis, in isolated, Langendorff-perfused *RyR2*^*p/s*^ and *RyR2*^*s/s*^ but not *WT* hearts. These effects were more marked at higher pacing frequencies and in the *RyR2*^*s/s*^ hearts. The latter showed the greatest arrhythmogenic tendency as well as extrasystolic events often followed by spontaneous sustained VT even during intrinsic beating. In addition to establishing an arrhythmogenic phenotype, and associating this with altered Ca^2+^ homeostasis, these data therefore associated a physiological CPVT phenotype with the clinically described *P2328S* mutation, in the cytoplasmic central region of the RyR2.

## Supporting information

We provide two sets of sequential images from isolated, Fluo-3-loaded myocytes as supplementary data that (1) provide further confirmation for the series of still images of propagating waves are shown in Fig. 5 that were obtained from *RyR2^s/s^* myocytes following treatment with isoproterenol and (2) distinguish them from the Ca^2+^ transients as obtained from *F*/*F*_0_ values obtained over fixed regions of interest, that were obtained from WT myocytes (Fig. 2b). These were made using frame captures of fluorescence (*F*) signals resulting from Ca^2+^ binding to fluo-3 excited by a 488-nm argon laser and detected between wavelengths of 505–550 nm sampled at 98 ms/frame (128 × 128 pixels/frame). The appropriateness of the chosen frame capture rate was corroborated in some experiments by confirming similar results following use of the faster line-scan mode with a sampling rate of 960 *μ*s/frame. The first series of images are from unstimulated *RyR2^s/s^* myocyte studied in the presence of isoproterenol. It illustrates a propagating increase of local Ca^2+^ fluorescence running along the cells concerned. This is in contrast to the second series of images from a regularly stimulated *WT* myocyte in which the evoked Ca^2+^ is synchronized over the whole cell surface.

Video Clip S1. (1)

Video Clip S2. (2)
